# ACE inhibitors in SSc patients display a risk factor for scleroderma renal crisis—a EUSTAR analysis

**DOI:** 10.1186/s13075-020-2141-2

**Published:** 2020-03-24

**Authors:** Lukas Bütikofer, Pierre A. Varisco, O. Distler, O. Kowal-Bielecka, Y. Allanore, G. Riemekasten, P. M. Villiger, S. Adler, on behalf of EUSTAR collaborators, Jérôme Avouac, Ulrich A. Walker, Serena Guiducci, Gabriele Riemekasten, Paolo Airò, Eric Hachulla, Gabriele Valentini, Patricia E. Carreira, Franco Cozzi, Alexandra Balbir Gurman, Yolanda Braun-Moscovici, Nemanja Damjanov, Lidia P. Ananieva, Raffaella Scorza, Sergio Jimenez, Joanna Busquets, Mengtao Li, Ulf Müller-Ladner, Britta Maurer, Alan Tyndall, Giovanni Lapadula, Florenzo Iannone, Radim Becvar, Stanislaw Sierakowsky, Otylia Kowal Bielecka, Maurizio Cutolo, Alberto Sulli, Giovanna Cuomo, Serena Vettori, Simona Rednic, Ileana Nicoara, P. Vlachoyiannopoulos, C. Montecucco, Roberto Caporali, Srdan Novak, László Czirják, Cecilia Varju, Carlo Chizzolini, Eugene J. Kucharz, Anna Kotulska, Magdalena Kopec-Medrek, Malgorzata Widuchowska, Blaz Rozman, Carmel Mallia, Bernard Coleiro, Armando Gabrielli, Dominique Farge, Adrian Hij, Roger Hesselstrand, Agneta Scheja, Frank Wollheim, Duska Martinovic, M. Govoni, Andrea Lo Monaco, Nicolas Hunzelmann, Raffaele Pellerito, Lisa Maria Bambara, Paola Caramaschi, Carol Black, Christopher Denton, Jörg Henes, Vera Ortiz Santamaria, Stefan Heitmann, Dorota Krasowska, Matthias Seidel, Mara Oleszowsky, Harald Burkhardt, Andrea Himsel, Maria J. Salvador, Bojana Stamenkovic, Aleksandra Stankovic, Mohammed Tikly, Maya N. Starovoytova, Merete Engelhart, Gitte Strauss, Henrik Nielsen, Kirsten Damgaard, Gabriella Szücs, Antonio Zea Mendoza, Carlos de la Puente Buijdos, Walter A. Sifuentes Giraldo, Øyvind Midtvedt, Torhild Garen, David Launay, Guido Valesini, Valeria Riccieri, Ruxandra Maria Ionescu, Daniela Opris, Laura Groseanu, Fredrick M. Wigley, Carmen M. Mihai, Roxana Sfrent Cornateanu, Razvan Ionitescu, Ana Maria Gherghe, Marilena Gorga, Rucsandra Dobrota, Mihai Bojinca, Georg Schett, Jörg H. W. Distler, Pierluigi Meroni, Silvana Zeni, Luc Mouthon, Filip De Keyser, Vanessa Smith, Francesco P. Cantatore, Ada Corrado, Susanne Ullman, Line Iversen, Maria R. Pozzi, Kilian Eyerich, Rüdiger Hein, Elisabeth Knott, Jacek Szechinski, Piotr Wiland, Magdalena Szmyrka-Kaczmarek, Renata Sokolik, Ewa Morgiel, Brigitte Krummel-Lorenz, Petra Saar, Martin Aringer, Claudia Günther, Branimir Anic, Marko Baresic, Miroslav Mayer, Sebastião C. Radominski, Carolina de Souza Müller, Valderílio F. Azevedo, Svetlana Agachi, Liliana Groppa, Lealea Chiaburu, Eugen Russu, Thierry Zenone, Simon Stebbings, John Highton, Lisa Stamp, Peter Chapman, Murray Baron, John O’Donnell, Kamal Solanki, Alan Doube, Douglas Veale, Marie O’Rourke, Esthela Loyo, Edoardo Rosato, Simonetta Pisarri, Cristina-Mihaela Tanaseanu, Monica Popescu, Alina Dumitrascu, Isabela Tiglea, Rodica Chirieac, Codrina Ancuta, Daniel E. Furst, Suzanne Kafaja, Paloma García de la Peña Lefebvre, Silvia Rodriguez Rubio, Marta Valero Exposito, Jean Sibilia, Emmanuel Chatelus, Jacques Eric Gottenberg, Hélène Chifflot, Ira Litinsky, Algirdas Venalis, Irena Butrimiene, Paulius Venalis, Rita Rugiene, Diana Karpec, Eduardo Kerzberg, Fabiana Montoya, Vanesa Cosentino, Ivan Castellvi

**Affiliations:** 1 CTU Bern and Institute of Social and Preventive Medicine (ISPM), University of Bern, Bern, Switzerland; 2 Swiss Health Insurance Service, Bern, Switzerland; 3 Department of Rheumatology, University Hospital Zurich, Zurich, Switzerland; 4 Medical University of Bialystok, Jana Kilińskiego 1, 15-089 Białystok, Poland; 5 Warsaw Medical University, Warsaw, Poland; 6 Cochin Hospital, Paris, France; 7 University Hospital Schleswig-Holstein, Lübeck, Germany; 8 Department of Rheumatology, Immunology and Allergology, University Hospital Bern, CH3010, Bern, Switzerland; 9 Department of Rheumatology, Helios Klinikum Erfurt, Erfurt, Germany

**Keywords:** Scleroderma renal crisis, ACE inhibitors, Arterial hypertension, Antihypertensive drugs, Outcome

## Abstract

**Objectives:**

To investigate the effect of ACE inhibitors (ACEi) on the incidence of scleroderma renal crisis (SRC) when given prior to SRC in the prospectively collected cohort from the European Scleroderma Trial and Research Group (EUSTAR).

**Methods:**

SSc patients without prior SRC and at least one follow-up visit were included and analyzed regarding SRC, arterial hypertension, and medication focusing on antihypertensive medication and glucocorticoids (GC).

**Results:**

Out of 14,524 patients in the database, we identified 7648 patients with at least one follow-up. In 27,450 person-years (py), 102 patients developed SRC representing an incidence of 3.72 (3.06–4.51) per 1000 py. In a multivariable time-to-event analysis adjusted for age, sex, disease severity, and onset, 88 of 6521 patients developed SRC. The use of ACEi displayed an increased risk for the development of SRC with a hazard ratio (HR) of 2.55 (95% confidence interval (CI) 1.65–3.95). Adjusting for arterial hypertension resulted in a HR of 2.04 (95%CI 1.29–3.24). There was no evidence for an interaction of ACEi and arterial hypertension (HR 0.83, 95%CI 0.32–2.13, *p* = 0.69). Calcium channel blockers (CCB), angiotensin receptor blockers (ARB), endothelin receptor antagonists, and GC—mostly in daily dosages below 15 mg of prednisolone—did not influence the hazard for SRC.

**Conclusions:**

ACEi in SSc patients with concomitant arterial hypertension display an independent risk factor for the development of SRC but are still first choice in SRC treatment. ARBs might be a safe alternative, yet the overall safety of alternative antihypertensive drugs in SSc patients needs to be further studied.

## Key messages

1. ACE inhibitors display a risk for the development of SRC in SSc patients
2. Arterial hypertension and ACE inhibitors are independent risk factors for SRC
3. Regarding SRC, alternative antihypertensive drugs need to be studied in SSc patients

## Introduction

ACE inhibitors (ACEi) are the mainstay of therapy in scleroderma renal crisis (SRC). Initiation of their use allowed for major increased survival rates of SRC over the last decades yet with mostly lasting sequelae [1]. Nevertheless, the incidence of SRC remained almost unchanged over the last decades and SRC risk factors are still poorly understood. Among those, the use of ACEi in hypertensive SSc patients prior to any SRC episode is meanwhile contradictorily discussed. While ACEi are supposed to lower the risk of SRC at the same time as they lower blood pressure, there are a few data that SRC outcome is worse in patients with prior ACEi intake.

A prospective online survey demonstrated a worse outcome in SRC patients when treated with ACEi prior to SRC onset [2]. On the other hand, pathophysiological reasoning cannot explain the phenomenon why a therapeutic agent should be discouraged when applied in a protective intention. The need for more valuable data is therefore often discussed among experts, but respective trials are rare, mostly retrospective, and difficult to conduct. One study by Guillevin et al. in as much as 91 patients pointed into the direction of favoring ACEi for SRC protection but was unable to draw firm conclusions [3].

Furthermore, besides some well-known factors for the risk of SRC, arterial hypertension is still discussed as bystander or promoting factor leaving the optimal choice for antihypertensive treatment unanswered.

Arterial hypertension in SSc patients might be present per se (i.e., independent from SSc itself) or at least in part due to hyperreninemia with activation of the renin-angiotensin-aldosterone system (RAAS)—which itself is discussed as a risk factor for SRC [4]. RAAS activation might therefore lead to the idea of initiating ACEi therapy.

The use of beta blockers and/or diuretics in the treatment of arterial hypertension in SSc patients is mostly precluded as they negatively affect the already reduced peripheral perfusion.

Calcium channel blockers (CCB) used to be the antihypertensive medication of choice in SSc patients due to their vasodilative effects and improvement in Raynaud’s phenomenon. Unfortunately, negative effects on, e.g., the esophagus, by smooth muscle relaxation [5] and sphincter pressures have been claimed although their clinical consequences on reflux and potential aspiration remain uncertain.

In the era of endothelin receptor antagonists (ERA) used for prevention of digital ulcers, the impact of CCBs at least in the indication for digital ulcer prevention might be vanishing. Furthermore, ERAs were supposed to be helpful in SRC as endothelin expression was shown to be high in kidney specimen from SRC patients [6]. Yet, they have only rarely been described in SRC case reports without convincing results [7].

The use of glucocorticoids (GC) especially in higher doses above 15 mg/d has long been known as important negative factor for the development of SRC, and the use of GC dosages above 15 mg/d is therefore discouraged [8]. However, the bias for indication cannot be ruled out in this setting with more severe patients having a higher propensity to receive high-dose corticosteroids. In addition, concomitant medication with, e.g., glucocorticoids in a situation of an activated RAAS additionally reduces renal flow by inhibiting synthesis of prostaglandins. Nevertheless, even recent data report on frequent if not routine use of GC in SSc patients for various reasons as, e.g., interstitial lung disease or progressive skin affection [9].

Data regarding incidence and influencing factors for SRC have and will always have limitations: they describe a very rare phenomenon in a rare disease with heterogeneous presentation and ongoing discussion about correct and/or consensus-driven diagnostic criteria [10]. The currently published data on possible classification criteria for SRC reflect this process [11]. The herein proposed core set of acute onset of hypertension, acute kidney injury, microangiopathic hemolytic anemia/thrombocytopenia, and target organ dysfunction is mainly consistent with the EUSTAR-based definition of SRC.

We therefore set out to analyze the European Scleroderma Trials and Research (EUSTAR) database representing the largest European and in part Extra-European prospective data collection from SSc patients, hereby focusing on ACEi, arterial hypertension, other anti-hypertensive medication, and glucocorticoids with respect to their influence on SRC.

## Methods

### Design

The EUSTAR database is a multicenter online database that contains prospectively collected data from more than 15,000 SSc patients in more than 200 international centers. Each patient’s annually scheduled visit for medical purposes is recorded providing longitudinal observational data. Each participating center has to obtain a positive ethics vote from their respective local ethical committee prior to including patients into the EUSTAR registry.

### Patients and medication

SSc patients were included at their first registered visit (referred to as baseline visit) if they had no reported SRC at or before this visit and at least one follow-up visit. Patients without any information about SRC or with missing visit dates were excluded.

Common EUSTAR definition of SRC is the abrupt onset of severe hypertension accompanied by rapidly progressive renal failure, hypertensive encephalopathy, congestive heart failure, and/or microangiopathic hemolytic anemia. Participating centers are expert centers only, and each EUSTAR center is trained by EUSTAR-specific courses including the definitions for disease entities as, e.g., SRC.

We used two different datasets: the so called “complete” dataset comprising all data within the EUSTAR database up to November 15, 2017, and a so called “medication” subset for which medication was consistently recorded—i.e., data collected at or after January 1, 2009, when definite documentation of medication within the EUSTAR database was started. In accordance with the complete dataset, the first visit after this date is referred to as baseline visit and patients with SRC before or at this baseline visit were excluded. For sensitivity analysis, the dataset was further reduced to patients enrolled in or after 2009.

The focus was laid on medication with ACEi, angiotensin receptor blockers (ARB), CCB, ERA, phospho-diesterase-5 (PDE5) inhibitors, and GC. The main analyses involving medication were based on the medication dataset.

### Statistical analysis

Patient characteristics are reported as median and interquartile range (IQR) or number and percentage of patients for continuous and categorical data, respectively. Patients with and without SRC were compared by Wilcoxon rank-sum tests and chi-squared tests, differences are presented as Hodges-Lehman median differences and risk differences with corresponding 95% confidence intervals (95% CI).

For survival analysis, patients were considered to be at risk after the baseline visit. The exact time point of SRC was interpolated between the last visit without and the first visit with renal crisis. Only visits up to the first SRC were considered. Patients without SRC were censored at the last registered visit. Missing time-varying covariates were handled by carrying the last observation forward. Patients with missing time-constant variables were excluded from the analysis.

Mortality is presented using a Kaplan-Meier failure plot, and the 5-year mortality was calculated using one minus the Kaplan-Meier estimator with the Greenwood pointwise standard error. Cumulative incidence of SRC was calculated using the Aalen-Johansson estimator with death (without SRC) as competing event.

We fitted cause-specific Cox proportional hazard models for SRC in which deaths were censored. Results are reported as hazard ratio (HR) with 95% CI. In sensitivity analyses, we used competing risk regressions with death (without SRC) as competing event to estimate sub-hazard ratios (sHR). Univariable models were fitted for a number of baseline and medication variables. All variables with a *p* value < 0.2 and age, sex, disease severity (whether or not there is diffuse skin involvement), and the time between onset of scleroderma and baseline visit were included in a multivariable analysis. Covariates were allowed to change over time if applicable. In sensitivity analyses, only values at baseline or at any time before SRC were used.

For further sensitivity analysis, we used propensity score methods to estimate the effect of ACEi at baseline or at any time before SRC on the hazard of SRC. Propensity scores were calculated from a logistic regression model for ACEi including the same set of covariates as the multivariable model. A common support was imposed by dropping treatment observations outside the range of the control propensity scores. Three different methods based on Stata command propensity score matching were used according to Leuven and Sianesi: one-to-one matching on the propensity score without replacement, k-nearest neighbors matching with replacement (with *k* = 3, *k* = 5, *k* = 10) and inverse probability weighting. Matching was performed with a caliper of 0.01. The matched observations were then used for a Cox regression with ACEi as the only covariate and robust standard errors to correct for the clustering based on the Huber-White sandwich estimator. For inverse probability weighting, 5% of the treatment observations at which the propensity score density of the control were lowest were dropped (trimming). We then calculated stabilized inverse probability weights and fitted a weighted Cox regression for ACEi as the only covariate [12].

For analysis of medication changes with focus on ACEi only patients in the medication dataset with SRC and visits before and after SRC were considered.

## Results

### Patient selection (Fig. 1)

Out of 14,524 eligible SSc patients, 9690 and 7648 patients were included in the complete and medication datasets, respectively. One hundred sixty-nine and 102 patients developed SRC in 45,071 and 27,450 person-years (py), representing an incidence per 1000 py of 3.75 (95% CI 3.22–4.36) and 3.72 (95% CI 3.06–4.51), respectively.

**Fig. 1:**
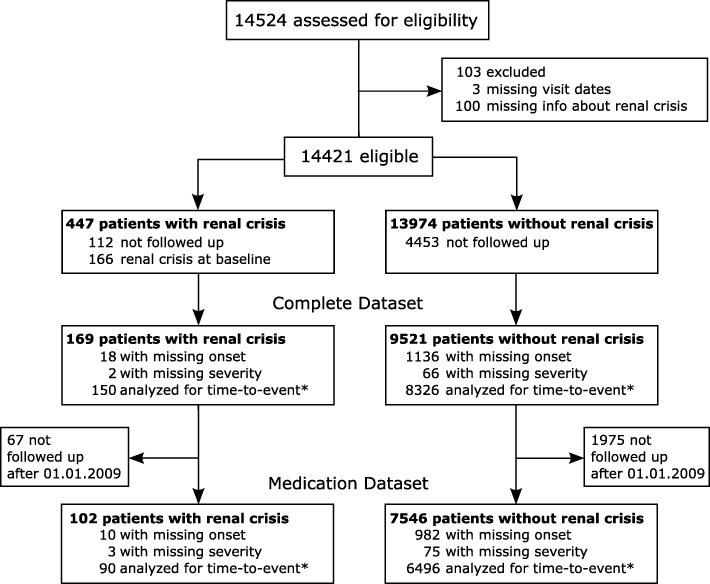
Patient flow. *Does not sum up as categories are not exclusive

In the complete dataset (supplementary Table 1), patients developing SRC over the course of the study were significantly more often male (40/169 vs 1292/9521, 24 vs 14%, *p* < 0.001) with a shorter disease duration (defined as onset of first non-Raynaud’s symptom) before inclusion in the study (3.1 vs 5.2 years, *p* < 0.001). At baseline, these patients displayed an SCL-70 antibody profile (72/158 vs 2962/8926, 46 vs 33%, *p* = 0.001) and demonstrated more often diffuse skin involvement (82/167 vs 2709/9267, 49 vs 29%, *p* < 0.001), arterial hypertension (63/166 vs 1862/9418, 38 vs 20%, *p* < 0.001), and tendon friction rubs (28/166 vs 722/9283, 17 vs 8%, *p* < 0.001), as well as muscle weakness and atrophy.

**Fig. 2:**
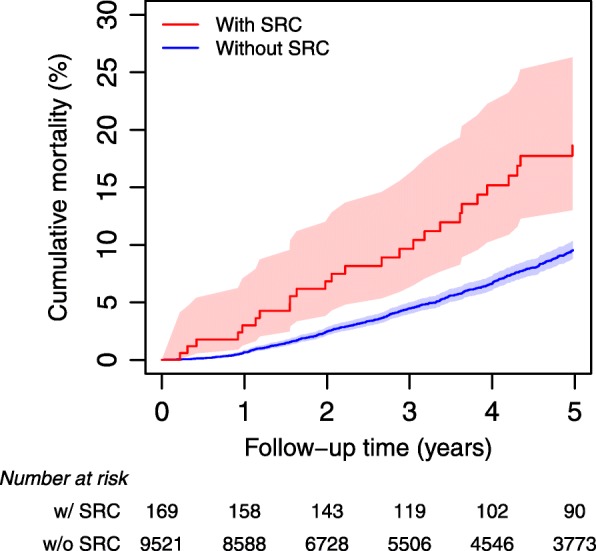
Kaplan-Meier failure plot for mortality with 95% confidence intervals of patients with and without scleroderma renal crisis (SRC) based on the complete dataset

Non-SRC patients were observed for a median time of 3.6 years (IQR 1.6 to 6.9) with 4 visits (IQR 2 to 6). Patients with SRC were observed for a median time of 5.0 years (IQR 2.5–8.4) with 6 visits (IQR 3–8). Median time to first onset of SRC was 1.7 years (IQR 0.5–4.2). Over the entire observation period, we documented death in patients with SRC (48/169) and without SRC (1025/9521, 28% vs 11%), leading to a 5-year mortality of 18.6% in SRC patients (95%CI 13.0–26.3%) and 9.5% (95%CI 8.8–10.3%) in non-SRC patients (Fig. 2).

### Patient characteristic medication dataset (Table 1)

Regarding medication, ACEi were given more often in patients who developed SRC over the course of the study (34/96 vs 1299/7163, 35 vs 18%, *p* < 0.001). For all other medication studied, we did not find any significant differences.

**Table 1:** Characteristics of medication dataset at baseline. Only the time up to the first SRC is considered

	Patients with SRC (*N* = 102)	Patients without SRC (*N* = 7546)	Median # or risk difference (95% CI)	*P* value
	*Median (IQR) or no. of patients (%)*			
Age (years)	57.3 (48.0 to 67.9)	56.4 (46.0 to 65.7)	1.3 (− 1.3 to 3.9)	0.36
Sex (female)	80/102 (78%)	6484/7546 (86%)	− 7% (− 16 to 1%)	0.031
Time between onset of scleroderma and inclusion (y)*	5.0 (1.9 to 10.9)	7.0 (3.1 to 13.2)	− 1.3 (− 2.5 to − 0.3)	0.019
Extent of skin involvement				0.005
No skin involvement	4/100 (4%)	306/7312 (4%)		
Only sclerodactyly	11/100 (11%)	785/7312 (11%)		
Limited cutaneous involvement	41/100 (41%)	4151/7312 (57%)		
Diffuse cutaneous involvement	44/100 (44%)	2070/7312 (28%)		
Glucocorticoids	37/90 (41%)	2437/6644 (37%)	4% (− 6 to 15%)	0.39
Dose at baseline (if > 0), mg	7.5 (5.0 to 10.0)	5.0 (5.0 to 10.0)	0.0 (0.0 to 2.0)	0.16
Glucocorticoids > 10 mg	5/90 (6%)	295/6644 (4%)	1% (− 4 to 6%)	0.61
Glucocorticoids > 15 mg	3/90 (3%)	171/6644 (3%)	1% (− 3 to 4%)	0.65
ACE inhibitors	34/96 (35%)	1299/7163 (18%)	17% (8 to 27%)	< 0.001
Angiotensin receptor blocker	6/96 (6%)	654/7150 (9%)	− 3% (− 8 to 2%)	0.33
Calcium channel blockers	50/96 (52%)	3763/7178 (52%)	− 0% (− 10 to 10%)	0.95
Endothelin receptor antagonist	12/85 (14%)	727/6165 (12%)	2% (− 5 to 10%)	0.51
PDE5 inhibitors	6/91 (7%)	321/6649 (5%)	2% (− 3 to 7%)	0.44

*Missing data for 10 patients with and 982 patients without SRC

^#^Generalized Hodges-Lehmann median differences

Median observation time was 3.1 years (1.5 to 5.6) for patients without SRC and 4.9 years (IQR 2.5–6.2) for patients that developed SRC. The first SRC was observed after a median of 1.5 years (IQR 0.5 to 3.4).

### Cumulative SRC incidence is negatively influenced by arterial hypertension and ACEi but not by CCB or GC

**Fig. 3:**
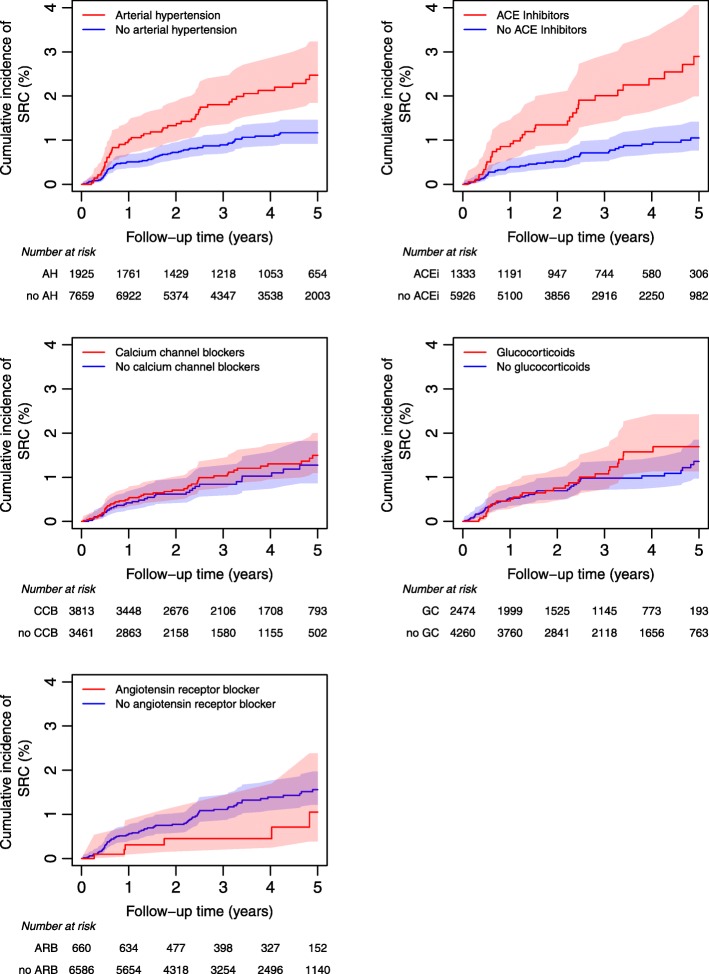
Cumulative incidence of SRC with 95% confidence intervals depending on whether patients have arterial hypertension (AH) based on the complete dataset or are treated with ACE inhibitors (ACE), calcium channel blockers (CCB), or glucocorticoids (GC) based on the medication dataset

Cumulative incidence of SRC was analyzed for the risk factors of interest, i.e., arterial hypertension, ACEi, CCB, GC, and ARB (Fig. 3). Death without SRC was treated as a competing event. Cumulative incidence for SRC was increased in patients treated with ACEi or suffering arterial hypertension but not for patients treated with CCB or GC. For the latter, a minor trend could be detected in the long-term application.

The effect of ACEi persisted in models adjusted for potential risk factors using propensity score matching or probability weighting (supplementary Fig. 1).

### Influencing factors for SRC

Potential risk factors for SRC were tested in univariable Cox proportional hazard models (supplementary Table 2) and included in a multivariable Cox model if evidence for an influence was found (Table 2). For the final model on the medication dataset, 6083 patients were included, and 78 experienced SRC.

**Table 2:** Hazard ratios for renal crisis from a multivariable Cox proportional hazard model based on the medication dataset. All variables with *p* < 0.2 in the univariable analyses were included

	No. of renal crises/patients	Hazard ratio (95% CI)	*P* value
Age (per decade)	78/6083	1.06 (0.87–1.28)	0.56
Sex (male)		1.30 (0.74–2.28)	0.36
Diffuse skin involvement		1.79 (1.06–3.02)	0.030
Time since onset of scleroderma (per decade)		0.77 (0.55–1.08)	0.13
Arterial hypertension		2.22 (1.34–3.66)	0.002
Tendon friction rub		1.70 (0.83–3.48)	0.15
ACE inhibitors		2.07 (1.28–3.36)	0.003
SCL70-positive		0.98 (0.58–1.66)	0.94
ACA-positive		0.82 (0.45–1.49)	0.52
Glucocorticoids > 10 mg		1.49 (0.53–4.17)	0.45
PDE5 inhibitors		1.32 (0.60–2.87)	0.49

An increased risk for SRC was found for diffuse skin involvement (hazard ratio (HR) 1.79, 95%CI 1.06–3.02, *p* = 0.030), arterial hypertension (HR 2.22, 95%CI 1.34–3.66, *p* = 0.002), and ACEi (HR 2.07, 95%CI 1.28–3.36, *p* = 0.003).

The results were largely confirmed in sensitivity analyses where time-varying variables were held constant by either using the baseline values (supplementary Table 3) or observation at any time over the follow-up period (supplementary Table 4), and if only patients enrolled in or after 2009 were analyzed (supplementary Table 5).

An alternative analysis using competing risk regression with death as competing event gave similar results with arterial hypertension and ACEi as most important risk factors (sHR 2.28 95%CI 1.36–3.81, *p* = 0.002 and sHR 2.07, 95% CI 1.27–3.38, *p* = 0.004, respectively) (supplementary Table 6).

Adjustment via propensity score matching or inverse probability weighting confirmed the effect of ACEi, regardless whether covariates at baseline or at any time over the course of the study were used (supplementary Table 7).

### Sensitivity analysis suggests ACEi and arterial hypertension as independent risk factors for SRC

We tested for an interaction of the two most important risk factors, arterial hypertension and ACEi, by adding an interaction term to the multivariable Cox proportional hazard model (Table 3). Evidence for an interaction was not found (HR of interaction term 0.83, 95%CI 0.32–2.13, *p* = 0.69) suggesting that ACEi and arterial hypertension were independent risk factors for SRC.

**Table 3:** Hazard ratios for SRC from a multivariable Cox proportional hazard model with an interaction of arterial hypertension and ACE inhibitors based on the medication dataset

	No. of renal crises/patients	Hazard ratio (95% CI)	*P* value
Age (per decade)	78/6083	1.06 (0.87–1.28)	0.58
Sex (male)		1.29 (0.74–2.27)	0.37
Diffuse skin involvement		1.78 (1.05–3.01)	0.032
Time since onset of scleroderma (per decade)		0.77 (0.55–1.08)	0.13
Arterial hypertension		2.41 (1.26–4.61)	0.008
Tendon friction rub		1.70 (0.83–3.48)	0.15
ACE inhibitors		2.28 (1.16–4.51)	0.018
SCL70-positive		0.98 (0.58–1.67)	0.95
ACA-positive		0.83 (0.46–1.50)	0.53
Glucocorticoids > 10 mg		1.49 (0.53–4.17)	0.45
PDE5 inhibitors		1.31 (0.60–2.86)	0.50
Arterial hypertension#ACE inhibitors		0.83 (0.32–2.13)	0.69

We also analyzed medication before and after SRC, i.e., assessed patients that received ACEi at any time point prior and after SRC. In most cases (49/69), ACEi were continued after renal crises.

## Discussion

Our work analyses the largest cohort of SSc patients with focus upon potentially influencing medication for the development of SRC.

To our surprise, ACEi independently and very prominently enhanced the hazard for SRC. Assuming that the main reason for the prescription of ACEi is arterial hypertension the latter was analyzed separately. We hereby wanted to rule out arterial hypertension itself as the main influence for SRC. Contrariwise ACEi and arterial hypertension proved independent risk factors and even more: both factors add up the risk for SRC. These results lead us to perform subanalyses regarding other potentially influencing factors finally confirming the initial results even more. In line with our main finding are the results of the QUINS trial [13]. Within this controlled trial, the ACE inhibition by long-term application of quinapril was not able to control vascular damage in SSc patients with limited cutaneous disease.

One of the questions is whether our cohort is different from other SRC cohorts. We found a SRC incidence of 3.72 (3.06–4.51) per 1000 py. Some of the first analyses by Steen et al. in 1984 could demonstrate an incidence of as high as 18% within their retrospective cohort of rapidly progressing SSc patients [14]. Since then SRC incidence considerably declined: In a recent large meta-analysis, an overall SRC prevalence of 4% was found during the last 30 years consistent with findings from the EUSTAR group describing a prevalence of 4.2% in the diffuse cutaneous (dc) SSc group [15, 16]. Mainly, most SRC risk factors as, e.g., dSSc, male sex, and rapid disease progression as displayed in our cohort are of course well-known risk factors for SRC [17, 18] with a longer duration of disease tending to reduce the risk of SRC as the counterpart. In addition, glucocorticoids given in higher dosages have already been described extensively as negatively influencing SRC incidence and outcome [19]. Here, our data could demonstrate a minimal effect of GC only, yet the average dosage was low with only 3% receiving more than 15 mg of prednisolone per day. So most physicians must have implemented this negative impact when deciding for immunosuppression of any kind. Furthermore, our data were collected prospectively in a standardized manner which allows for adequate documentation with only few missings.

Given a thorough analysis of a well-defined, prospectively collected cohort, what reasoning might at least in part explain our findings? Possibly, in SSc patients with long standing ACEi therapy, an aldosterone breakthrough mechanism with elevated aldosterone and renin levels therapy might come into account [20] leading to further unwanted vasoconstriction and endothelial cell proliferation. In this case, direct renin inhibition seems to be an option. Yet, this inhibition was not able to prevent from breakthrough mechanisms so far [21]. Furthermore, renin inhibition has only infrequently been used in SSc patients [22] which is as well reflected in our analysis as no EUSTAR patient had received renin inhibitors.

Therefore, regarding angiotensin II, its direct blockade appears to be an option, which might guide therapy into the direction of ARBs. This hypothesis is clearly supported by our data: as more than 600 patients were treated with ARBs, we judge the results valuable: In clear contrast to the use of ACEi, ARBs demonstrated a slightly positive effect on the hazard of SRC and definitely no negative effect at all.

Other medication analyzed within our cohort had a neutral effect on SRC occurrence as, e.g., CCBs: they showed no additional influence in concomitant arterial hypertension. This is in agreement with a recent analysis of the Canadian Scleroderma Research Group, which did not find any association of CCB with SRC [23].

Secondly, some medication was given in few patients only. ERA for example might have some indication in SRC treatment as ET receptors can be expressed within SRC [24] and rare cases with positive effects of ET receptor blocking therapy in SRC have been observed [25]. No data exist on their preventive potential and our study does not contribute enough evidence to allow for a clear description of any influence on SRC incidence in either way.

Overall, our data clearly demonstrate an increased risk for SRC when ACEi are used in SSc patients prior to the onset of any SRC. Nevertheless, the significant and independent negative influence of arterial hypertension on the risk of SRC demands antihypertensive treatment. Unfortunately, the list of acceptable antihypertensive medications in SSc patients is short and—after removal of ACEi—mostly CCB and ARBs are left. The large number of SSc patients with ARB treatment in our cohort without a negative signal on SRC incidence might make it a valuable alternative to ACEi at present. We hope that a more frequent use of newer antihypertensive drugs in the future will broaden our understanding of their potential influence and safety regarding SRC in hypertensive SSc patients.

### Strengths and limitations

The EUSTAR database is the largest systematic and prospective data collection regarding SSc and SRC.

In order to obtain reliable and consistent information, we had to reduce the dataset considerably. Most notably, we had to restrict the analysis of the medication on a subset for which the documentation was consistent. Furthermore, the main patient characteristics were not changed and the analysis of all variables beside medication showed similar results. As to the nature of the database indications for a specific treatment, e.g., ACEi cannot be specified. Nevertheless, as ACEi remain among the most frequently used antihypertensive drugs, the conclusion to analyze its influence in relation to arterial hypertension appears justified.

The measure of both outcome and exposure was imprecise and not available on the same regular basis for all patients. Furthermore, as the number of visits and the time span of EUSTAR-documented visits differ between patients with and without SRC, time-varying exposure variables may not be observed with the same likelihood. However, the most important findings were shown to be stable within different definitions of the exposure (time-varying versus baseline and anytime).

Antibodies to RNA polymerase III are known to be associated with SRC. Unfortunately, their measurement has not been a routine procedure in most of the associated EUSTAR centers so far. Therefore, we were not able to give additional information about RNA polymerase III antibodies and their link to SRC as discussed here but this should clearly be addressed in future studies.

## Conclusion

ACEi in SSc patients with concomitant arterial hypertension display an independent risk factor for the development of SRC. Still, they are the mainstay of treatment once SRC is established. ARBs might be a safe option in the treatment of arterial hypertension with a possibly lower risk for development of SRC. Yet, the overall safety of alternative antihypertensive drugs in SSc patients needs to be studied.

## Supplementary information

**Additional file 1.**

**Additional file 2: Table S1.** Characteristics of patients in the complete dataset at baseline. Only the time up to the first SRC is considered.

**Additional file 3: Figure S1.** Cumulative incidence of SRC depending on whether patients are treated with ACE inhibitors at baseline (A) or at any time before SRC (B), adjusted using one-to-one propensity score matching (left panels) or inverse probability weighting (right panel). Propensity score were modeled using age, sex, disease severity, and time since onset of scleroderma at baseline, and arterial hypertension, tendon friction rub, SCL70, ACA, glucocorticoids > 10 mg and PDE5 inhibitors measured at baseline (A) or at any time before SRC (B).

**Additional file 4: Table S1.** Hazard ratios for renal crisis from univariable Cox proportional hazard models based on (A) the complete and (B) the medication dataset.

**Additional file 5: Table S2.** Hazard ratios for renal crisis from a multivariable Cox proportional hazard model with covariates measured at baseline based on the medication dataset.

**Additional file 6: Table S3.** Hazard ratios for renal crisis from a multivariable Cox proportional hazard model with covariates observed at any time before renal crisis based on the medication dataset.

**Additional file 7: Table S4.** Hazard ratios for renal crisis from a multivariable Cox proportional hazard model when only patients enrolled after 01.01.2009 are considered (i.e. the reduced medication dataset).

**Additional file 8: Table S5.** Subhazard ratios for renal crisis from a multivariable competing risk model with death (without SRC) as competing event based on the medication dataset.

**Additional file 9: Table S6.** Hazard ratios for the effect of ACEi on SRC from Cox proportional hazard models adjusted for age, sex, disease severity, and time since onset of scleroderma at baseline, and arterial hypertension, tendon friction rub, SCL-70, ACA, glucocorticoids >10mg and PDE5 inhibitors measured at baseline or at any time before renal crisis using different propensity score methods, i.e. one-to-one matching, k-nearest neighbors matching and inverse probability weighting.

## Data Availability

The datasets analyzed during the current study are available from the corresponding author upon reasonable request.
