# Supplementary material for: ACE inhibitors in SSc patients display a risk factor for scleroderma renal crisis—a EUSTAR analysis
Source: Arthritis Res Ther. 2020 Mar 24;22:59. doi: 10.1186/s13075-020-2141-2 (PMC7093969; doi:10.1186/s13075-020-2141-2)
Supplement: Supplementary file 1 — Additional file 1. [file 13075_2020_2141_MOESM1_ESM.docx]

EUSTAR co-authors:

Jérôme Avouac (1), Ulrich A. Walker (2), Serena Guiducci (3), Gabriele Riemekasten (4), Paolo Airò (5), Eric Hachulla (6), Gabriele Valentini (7), Patricia E. Carreira (8), Franco Cozzi (9), Alexandra Balbir Gurman (10), Yolanda Braun-Moscovici (10), Nemanja Damjanov (11), Lidia P. Ananieva (12), Raffaella Scorza (13), Sergio Jimenez (14), Joanna Busquets (14), Mengtao Li (15), Ulf Müller-Ladner (16), Britta Maurer (17), Alan Tyndall (18), Giovanni Lapadula (19), Florenzo Iannone (19), Radim Becvar (20), Stanislaw Sierakowsky (21), Otylia Kowal Bielecka (21), Maurizio Cutolo (22), Alberto Sulli (22), Giovanna Cuomo (7), Serena Vettori (7), Simona Rednic (23), Ileana Nicoara (23), P. Vlachoyiannopoulos (24), C. Montecucco (25), Roberto Caporali (25), Srdan Novak (26), László Czirják (27); Cecilia Varju (27), Carlo Chizzolini (28), Eugene J. Kucharz (29), Anna Kotulska (29), Magdalena Kopec-Medrek (29), Malgorzata Widuchowska (29), Blaz Rozman (30), Carmel Mallia (31), Bernard Coleiro (31), Armando Gabrielli (32), Dominique Farge (33), Adrian Hij (33), Roger Hesselstrand (34), Agneta Scheja (34), Frank Wollheim (34), Duska Martinovic (35), M. Govoni (35), Andrea Lo Monaco (36), Nicolas Hunzelmann (37), Raffaele Pellerito (38), Lisa Maria Bambara (39), Paola Caramaschi (39), Carol Black (40), Christopher Denton (40), Jörg Henes (41), Vera Ortiz Santamaria (42), Stefan Heitmann (43), Dorota Krasowska (44), Matthias Seidel (45), Mara Oleszowsky (45), Harald Burkhardt (46), Andrea Himsel (46), Maria J. Salvador (47), Bojana Stamenkovic (48), Aleksandra Stankovic (48), Mohammed Tikly (49), Maya N. Starovoytova (50), Merete Engelhart (51), Gitte Strauss (51), Henrik Nielsen (51), Kirsten Damgaard (51), Gabriella Szücs(52), Antonio Zea Mendoza (52), Carlos de la Puente Buijdos (53), Walter A. Sifuentes Giraldo (53), Øyvind Midtvedt (54), Torhild Garen (54), David Launay (6), Guido Valesini (55), Valeria Riccieri (55), Ruxandra Maria Ionescu (56), Daniela Opris (56), Laura Groseanu (56), Fredrick M. Wigley (57), Carmen M. Mihai (58), Roxana Sfrent Cornateanu (58), Razvan Ionitescu (58), Ana Maria Gherghe (58), Marilena Gorga (58), Rucsandra Dobrota (58), Mihai Bojinca (58) , Georg Schett (59), Jörg HW Distler (59), Pierluigi Meroni (60), Silvana Zeni (60), Luc Mouthon (61), Filip De Keyser (62), Vanessa Smith (62), Francesco P. Cantatore (63), Ada Corrado (63), Susanne Ullman (64), Line Iversen (64), Maria R. Pozzi (65), Kilian Eyerich (66), Rüdiger Hein (66), Elisabeth Knott (66), Jacek Szechinski (67), Piotr Wiland (67), Magdalena Szmyrka-Kaczmarek (67), Renata Sokolik (67), Ewa Morgiel (67), Brigitte Krummel-Lorenz (68), Petra Saar (69), Martin Aringer (69), Claudia Günther (69), Branimir Anic (70), Marko Baresic (70), Miroslav Mayer (70), Sebastião C. Radominski (71), Carolina de Souza Müller (71), Valderílio F. Azevedo (71), Svetlana Agachi (72), Liliana Groppa (72), Lealea Chiaburu (72), Eugen Russu (72), Thierry Zenone (73), Simon Stebbings (74), John Highton (74), Lisa Stamp (75), Peter Chapman (75), Murray Baron (76), John O'Donnell (76), Kamal Solanki (77), Alan Doube (77), Douglas Veale (78), Marie O'Rourke (78), Esthela Loyo (79), Edoardo Rosato (80), Simonetta Pisarri (81), Cristina-Mihaela Tanaseanu (82), Monica Popescu (82), Alina Dumitrascu (82), Isabela Tiglea (82), Rodica Chirieac (82), Codrina Ancuta (82), Daniel E. Furst (83), Suzanne Kafaja (83), Paloma García de la Peña Lefebvre (84), Silvia Rodriguez Rubio (84), Marta Valero Exposito (84), Jean Sibilia (85), Emmanuel Chatelus (85), Jacques Eric Gottenberg (85), Hélène Chifflot (85), Ira Litinsky (86), Algirdas Venalis (87), Irena Butrimiene (87), Paulius Venalis (87), Rita Rugiene (87), Diana Karpec (87), Eduardo Kerzberg (88), Fabiana Montoya (88), Vanesa Cosentino (88), Ivan Castellvi (89).

# (1) Rheumatology A, Paris Descartes University, Cochin Hospital, Paris, France, (2) Department of Rheumatology, Basel University, Unispital Basel, Switzerland, (3) Department of Experimental and Clinical Medicine, Section of Internal Medicine and Division of Rheumatology, Azienda Ospedaliero-Universitaria Careggi (AOUC), University of Florence, Florence, Italy , (4) Department of Rheumatology, Charitè University Hospital, Berlin, German Rheumatism Research Centre Berlin (DRFZ), a Leibniz institute, Germany, (5) Spedali Civili di Brescia, Servizio di Reumatologia Allergologia e Immunologia Clinica, Brescia, Italy, (6) Department of Internal Medicine, Hôpital Claude Huriez, Lille cedex, France, (7) Department of Clinical and Experimental Medicine “F-Magrassi” II, Naples, Italy, (8) Servicio de Reumatología, Hospital 12 de Octubre, Madrid, Spain , (9) Rheumatology Unit, Department of Clinical and Experimental Medicine, University of Padova, Italy, (10) B. Shine Department of Rheumatology, Rambam Health Care Campus, Haifa, Israel, (11) Institute of Rheumatology, Belgrade, Serbia & Montenegro, (12) Institute of Rheumatology, Russian Academy of Medical Science, Moscow, Russia, (13) U.O. Immunologia Clinica - Centro di Riferimento per le Malattie Autoimmuni Sistemiche, Milano, Italy, (14) Thomas Jefferson University, Philadelphia, Pennsylvania, USA, (15) Department of Rheumatology, Peking Union Medical College Hospital (West Campus), Chinese Academy of Medical Sciences, Beijing, China, (16) Department of Rheumatology and Clinical Immunology, Justus-Liebig University Giessen, Kerckhoff Clinic, Bad Nauheim, Germany, (17) Department of Rheumatology, University Hospital Zurich, Zurich, Switzerland (18) Department of Rheumatology, University Hospital Basel, Switzerland, (19) Rheumatology Unit-DiMIMP, School of Medicine University of Bari, Italy, (20) Institute of Rheumatology, 1st Medical School, Charles University, Prague, Czech Republic, (21) Department of Rheumatology and Internal Diseases, Medical University of Bialystok, Poland, (22) Research Laboratory and Division of Rheumatology Department of Internal Medicine, University of Genova, Italy, (23) Department of Rheumatology, University of Medicine & Pharmacy "Iuliu Hatieganu" Cluj, Cluj-Napoca, Romania, (24) Department of Pathopysiology, Medical School, National University of Athens, Greece, (25) Unita' Operativa e Cattedra di Reumatologia, IRCCS Policlinico S Matteo, Pavia, Italy, (26) Department of Rheumatology and Clinical Immunology, Internal Medicine, KBC Rijeka, Croatia, (27) Department of Immunology and Rheumatology, Faculty of Medicine, University of Pécs, Hungary, (28) Department of Immunology and Allergy, University Hospital, Geneva, Switzerland, (29) Department of Internal Medicine and Rheumatology, Medical University of Silesia, Katowice, Poland, (30) University Medical Center Ljublijana, Division of Internal Medicine, Department of Rheumatology, Ljubliana, Slovenia, (31) “Stella Maris”, Balzan, Malta, (32) Istituto di Clinica Medica Generale, Ematologia ed Immunologia Clinica, Università Politecnica delle Marche Polo Didattico, University of Ancona, Italy, (33) Assistance Publique-Hôpitaux de Paris; Saint-Louis Hospital; Internal Medicine and Vascular Disease Unit ; INSERM UMRS 1160; Paris 7 Diderot University, Sorbonne Paris Cité, 1 avenue Claude-Vellefaux, 75010 Paris, France, (34) Department of Rheumatology, Lund University Hospital, Sweden, (35) Department of Internal Medicine, Clinical Hospital of Split, Croatia, (36) Department of Clinical and Experimental Medicine, Rheumatology Unit, University of Ferrara, Italy, (37) Department of Dermatoloy, University Hospital Cologne, Germany, (38) Ospedale Mauriziano, Centro di Reumatologia, Torino, Italy, (39) Università degli Studi di Verona, Dipartimento di Medicina Clinica e Sperimentale, Reumatologia-Medicina Interna B, Policlinico GB Rossi, Verona, Italy, (40) Centre for Rheumatology, Royal Free and University College London Medical School, London, United Kingdom, (41) Medizinische Universitätsklinik, Abt. II (Onkologie, Hämatologie, Rheumatologie, Immunologie, Pulmonologie), Tübingen, Germany, (42) Rheumatology Granollers General Hospital, Barcelona, Spain, (43) Department of Rheumatology, Marienhospital Stuttgart, Germany, (44) Department of Dermatology, Medical University of Lublin, Poland, (45) Medizinische Universitäts-Poliklinik, Department of Rheumatology, Bonn, Germany, (46) Klinikum der Johann Wolfgang Goethe Universität, Medizinische Klinik III, Rheumatologische Ambulanz, Frankfurt am Main, Germany, (47) Rheumatology Department, Hospitais da Universidade, Coimbra, Portugal, (48) Institute for Prevention, Treatment and Rehabilitation of Rheumatic and Cardiovascular Diseases, Niska Banja, Serbia and Montenegro, (49) Rheumatology Unit, Department of Medicine Chris Hani Haragwanath, Hospital and University of the Witwatersrand, Johannesburg, South Africa, (50) Institute of Rheumatology, 1st Medical School, Charles University, Prague, Czech Republic, (51) Department of Rheumatology, University Hospital of Gentofte, Hellerup, Denmark, (52)Third Department of Medicine, Rheumatology Division, University of Debrecen, Medical Center, Debrecen, Hungary, (53) Servicio de Reumatología, Hospital Ramon Y Cajal, Madrid, Spain, (54) Department of Rheumatology, Rikshospitalet University Hospital, Oslo, Norway, (55) Department of Internal Medicine and Medical Specialities, “Sapienza” University of Rome, Italy, (56) Department of Rheumatology - St. Maria Hospital, Carol Davila, University of Medicine and Pharmacy, Bucharest, Romania, (57) Johns Hopkins University, Division of Rheumatology, Johns Hopkins School of Medicine, Baltimore, USA,(58) Department of Internal Medicine and Rheumatology Clinic, Ion Cantacuzino Clinical Hospital, Bucharest, Romania, (59) Department of Internal Medicine 3, University Hospital Erlangen, Germany, (60) Dipartimento e Cattedra di Reumatologia, Università degli Studi di Milano, Istituto Ortopedico "Gaetano Pini", Milano, Italy, (61) Department of Internal Medicine, Hôpital Cochin, Paris, France, (62) University of Ghent, Department of Rheumatology, Gent, Belgium, (63) U.O. Reumatologia-Università degli Studi di Foggia, Ospedale "Col. D'Avanzo", Foggia, Italy, (64) University Hospital of Copenhagen, Department of Dermatology D-40, S-Bispebjerg Hospital, Copenhagen, Denmark, (65) Dipartimento di Medicina, Ospedale San Gerardo, Monza, Italy, (66) Department of Dermatology and Allergy of the TU Munich, Germany, (67) Department of Rheumatology and Internal Diseases, Wroclaw University of Medicine, Wroclaw, Poland, (68) Endokrinologikum Frankfurt, Germany, (69) Division of Rheumatology, Department of Medicine III/Department of Dermatology, University Medical Center Carl Gustav Carus, Technical University of Dresden, Germany, (70) University Hospital Centre Zagreb, Division of Clinical Immunology and Rheumatology, Department of Medicine, Zagreb, Croatia, (71) Hospital de Clínicas da Universidade Federal do Paraná, Curitiba - Paraná, Brasil, (72) Municipal Centres of Research in Scleroderma, Hospital "Sacred Trinity", Department of Rheumatology, Chisinau, Republic of Moldova, (73) Department of Medicine, Unit of Internal Medicine, Valence cedex 9, France, (74) Dunedin School of Medicine, Dunedin, New Zealand, (75) Department of Medicine, University of Otago, Christchurch, New Zealand, (76) Murray Baron, McGill University, Jewish General Hospital, Montreal, Quebec, Canada, (77) Waikato University Hospital, Rheumatology Unit, Hamilton City, New Zealand, (78) Department of Rheumatology, Bone and Joint Unit, St. Vincent’s University Hospital, Dublin, Ireland, (79) Reumatologia e Inmunologia Clinica, Hospital Regional Universitario Jose Ma Cabral y Baez, Clinica Corominas, Santiago, Dominican Republic, (80) Centro per la Sclerosi Sistemica - Dipartimento di Medicina Clinica, Università La Sapienza, Policlinico Umberto I, Roma, Italy, (81) Clinical Emergency Hospital St. Pantelimon, Bucharest, Romania, (82) Division of Rheumatology & Rehabilitation GR.T.Popa, Center for Biomedical Research, European Center for Translational Research-"GR.T.Popa" University of Medicine and Pharmacy, Rehabilitation Hospital, Iasi, Romania, (83) Division of Rheumatology, Department of Medicine, University of California at Los Angeles, Rehab Center, Los Angeles, USA, (84) Hospital Universitario Madrid Norte Sanchinarro, Madrid, Spain, (85) University Hospital of Strasbourg-Department of Rheumatology, Hôpital de Hautepierre, Service de Rhumatologie, Strasbourg Cedex, France, (86) Department of Rheumatology, Tel-Aviv Sourasky Medical Center, Tel-Aviv, Israel, (87) State Research Institute for Innovative Medicine, Vilnius University, Vilnius, Lithuania, (88) Osteoarticular Diseases and Osteoporosis Centre, Pharmacology and Clinical Pharmacological Research Centre, School of medicine - University of Buenos Aires, Rheumatology and Collagenopathies Department, Ramos Mejía Hospital, Buenos Aires, Argentina, (89) Hospital de la Santa Creu i Sant Pau, Barcelona, Catalunya, Spain.
