# Supplementary material for: ACE inhibitors in SSc patients display a risk factor for scleroderma renal crisis—a EUSTAR analysis
Source: Arthritis Res Ther. 2020 Mar 24;22:59. doi: 10.1186/s13075-020-2141-2 (PMC7093969; doi:10.1186/s13075-020-2141-2)
Supplement: Supplementary file 2 — Additional file 2: Table S1. Characteristics of patients in the complete dataset at baseline. Only the time up to the first SRC is considered. [file 13075_2020_2141_MOESM2_ESM.docx]

supplementary table 1: Characteristics of patients in the complete dataset at baseline. Only the time up to the first SRC is considered.

|  | Patients with SRC (N=169) | Patients without SRC (N=9521) | | Median# or risk difference (95% CI) | P value | | | |
| --- | --- | --- | --- | --- | --- | --- | --- | --- |
|  | *median (IQR) or no. of patients (%)* | |  |  |  | | | |
| Age (y) | 57.0 (47.1 to 67.3) | 55.3 (45.0 to 64.5) | | 1.9 (-0.3 to 4.0) | 0.09 | | | |
| Sex (female) | 129/169 (76%) | 8229/9521 (86%) | | -10% (-17 to -4%) | <0.001 | | | |
| Time between onset of scleroderma and inclusion (y)* | 3.1 (1.2 to 9.6) | 5.2 (2.0 to 11.1) | | -1.1 (-1.8 to -0.6) | <0.001 | | | |
| Extent of skin involvement |  |  | |  | <0.001 | | | |
| no skin involvement | 4/167 (2%) | 306/9267 (3%) | |  |  | | | |
| only sclerodactyly | 16/167 (10%) | 972/9267 (10%) | |  |  | | | |
| limited cutaneous involvement | 65/167 (39%) | 5280/9267 (57%) | |  |  | | | |
| diffuse cutaneous involvement | 82/167 (49%) | 2709/9267 (29%) | |  |  | | | |
| Arterial hypertension | 63/166 (38%) | 1862/9418 (20%) | | 18% (11 to 26%) | <0.001 | | | |
| Esophagal symptoms | 110/168 (65%) | 5987/9441 (63%) | | 2% (-5 to 9%) | 0.58 | | | |
| Stomach symptoms | 40/168 (24%) | 2069/9397 (22%) | | 2% (-5 to 8%) | 0.58 | | | |
| Intestinal symptoms | 33/167 (20%) | 2189/9418 (23%) | | -3% (-10 to 3%) | 0.29 | | | |
| Palpitations | 37/164 (23%) | 2034/9279 (22%) | | 1% (-6 to 7%) | 0.84 | | | |
| Joint contracture | 60/164 (37%) | 2459/9332 (26%) | | 10% (3 to 18%) | 0.003 | | | |
| Tendon friction rub | 28/166 (17%) | 722/9283 (8%) | | 9% (3 to 15%) | <0.001 | | | |
| Muscle weakness | 51/167 (31%) | 1813/9336 (19%) | | 11% (4 to 18%) | <0.001 | | | |
| Muslce atrophy | 29/166 (17%) | 765/9301 (8%) | | 9% (3 to 15%) | <0.001 | | | |
| ANA positive | 156/163 (96%) | 8772/9277 (95%) | | 1% (-2 to 4%) | 0.52 | | | |
| ACA positive | 39/157 (25%) | 3450/8914 (39%) | | -14% (-21 to -7%) | <0.001 | | | |
| SC170 positive | 72/158 (46%) | 2962/8926 (33%) | | 12% (5 to 20%) | 0.001 | | | |
| RNA polymerase III positive | 2/36 (6%) | 137/2864 (5%) | | 1% (-7 to 8%) | 0.83 | | | |
| ACR criteria fullfilled | 143/165 (87%) | 7735/9324 (83%) | | 4% (-2 to 9%) | 0.21 | | | |
| *Missing data for 18 patients with and 1136 patients without SRC | | | | |  |  |  |  |
| #Generalized Hodges-Lehmann median differences | | | | |  |  |  |  |
