# Supplementary figures and images for: ACE inhibitors in SSc patients display a risk factor for scleroderma renal crisis—a EUSTAR analysis
Source: Arthritis Res Ther. 2020 Mar 24;22:59. doi: 10.1186/s13075-020-2141-2 (PMC7093969; doi:10.1186/s13075-020-2141-2)

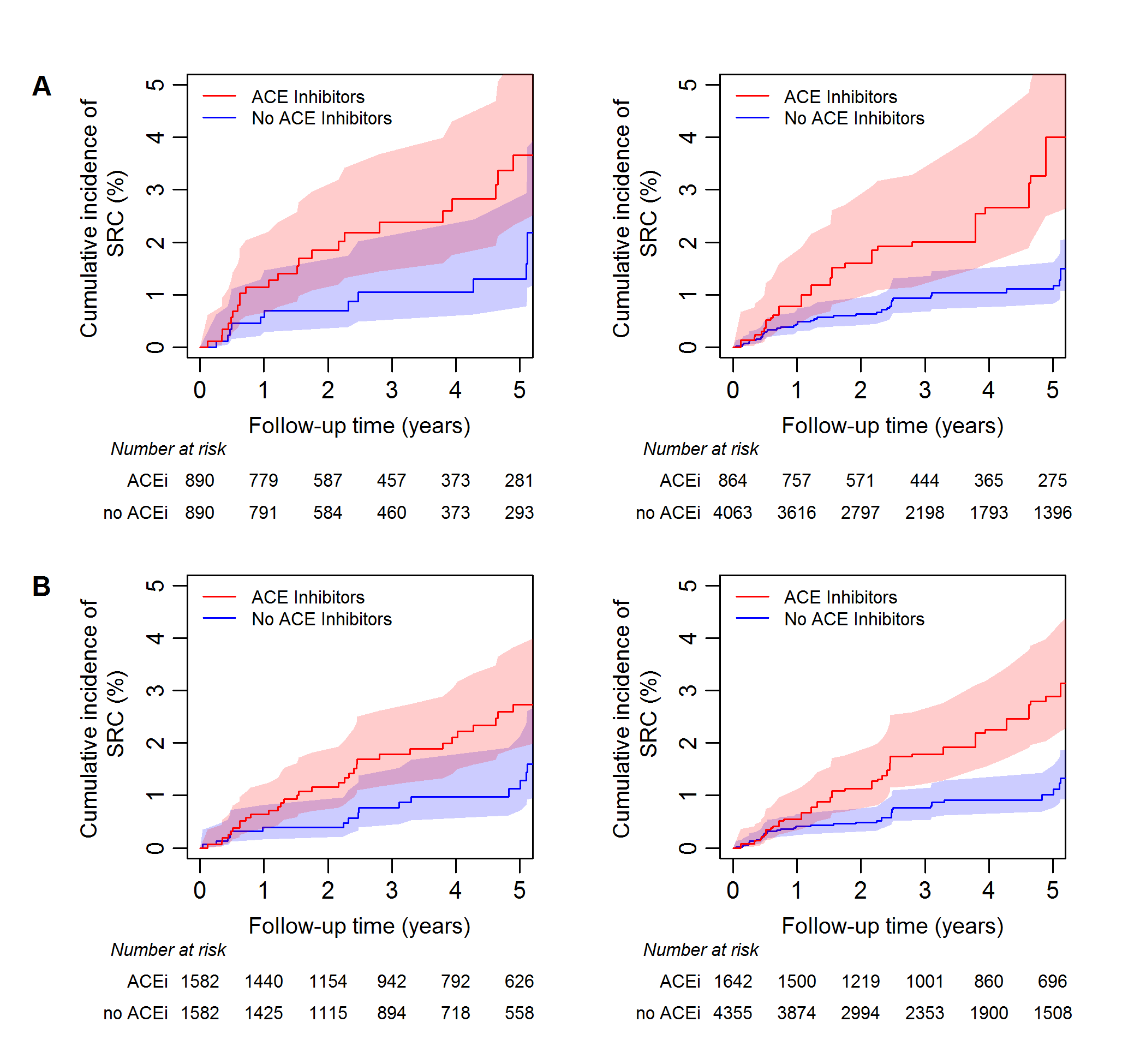

Supplement: Supplementary file 3 — Additional file 3: Figure S1. Cumulative incidence of SRC depending on whether patients are treated with ACE inhibitors at baseline (A) or at any time before SRC (B), adjusted using one-to-one propensity score matching (left panels) or inverse probability weighting (right panel). Propensity score were modeled using age, sex, disease severity, and time since onset of scleroderma at baseline, and arterial hypertension, tendon friction rub, SCL70, ACA, glucocorticoids > 10 mg and PDE5 inhibitors measured at baseline (A) or at any time before SRC (B). [file 13075_2020_2141_MOESM3_ESM.docx]
