# Supplementary material for: ACE inhibitors in SSc patients display a risk factor for scleroderma renal crisis—a EUSTAR analysis
Source: Arthritis Res Ther. 2020 Mar 24;22:59. doi: 10.1186/s13075-020-2141-2 (PMC7093969; doi:10.1186/s13075-020-2141-2)
Supplement: Supplementary file 4 — Additional file 4: Table S1. Hazard ratios for renal crisis from univariable Cox proportional hazard models based on (A) the complete and (B) the medication dataset. [file 13075_2020_2141_MOESM4_ESM.docx]

|  | No. of renal crises/patients | Hazard ratio (95% CI) | P value |
| --- | --- | --- | --- |
| **A** |  |  |  |
| Age (per decade) | 169/9690 | 1.16 (1.04 - 1.31) | 0.011 |
| Sex (male) | 169/9690 | 2.15 (1.50 - 3.06) | <0.001 |
| Diffuse skin involvement | 167/9622 | 2.56 (1.89 - 3.46) | <0.001 |
| Time since onset of scleroderma (per decade) | 151/8536 | 0.73 (0.57 - 0.94) | 0.015 |
| Arterial hypertension | 167/9684 | 2.54 (1.86 - 3.46) | <0.001 |
| Tendon friction rub | 167/9653 | 3.20 (2.14 - 4.78) | <0.001 |
| ACA positive | 161/9477 | 0.51 (0.35 - 0.73) | <0.001 |
| SCL70 positive | 161/9477 | 1.56 (1.14 - 2.13) | 0.005 |
| **B** |  |  |  |
| Age (per decade) | 102/7648 | 1.09 (0.94 - 1.27) | 0.24 |
| Sex (male) | 102/7648 | 1.81 (1.13 - 2.90) | 0.014 |
| Diffuse skin involvement | 99/7570 | 2.16 (1.45 - 3.21) | <0.001 |
| Time since onset of scleroderma (per decade) | 92/6656 | 0.74 (0.55 - 1.00) | 0.05 |
| Arterial hypertension | 99/7625 | 2.59 (1.74 - 3.86) | <0.001 |
| Tendon friction rub | 98/7583 | 2.03 (1.05 - 3.90) | 0.034 |
| ACA positive | 93/7345 | 0.53 (0.33 - 0.84) | 0.007 |
| SCL70 positive | 93/7368 | 1.39 (0.92 - 2.11) | 0.12 |
| ACE inhibitors | 99/7573 | 2.66 (1.78 - 3.98) | <0.001 |
| Glucocorticoids | 96/7449 | 1.08 (0.72 - 1.62) | 0.72 |
| Glucocorticoids > 10mg | 96/7449 | 2.06 (0.84 - 5.07) | 0.12 |
| Glucocorticoids > 15mg | 96/7449 | 1.61 (0.40 - 6.52) | 0.51 |
| Angiotensin receptor blocker | 99/7579 | 0.74 (0.36 - 1.52) | 0.41 |
| Calcium channel blockers | 99/7590 | 1.01 (0.68 - 1.51) | 0.94 |
| Endothelin receptor antagonist | 93/7432 | 1.37 (0.81 - 2.33) | 0.24 |
| PDE5 inhibitors | 95/7468 | 1.61 (0.84 - 3.11) | 0.15 |
