# Supplementary material for: ACE inhibitors in SSc patients display a risk factor for scleroderma renal crisis—a EUSTAR analysis
Source: Arthritis Res Ther. 2020 Mar 24;22:59. doi: 10.1186/s13075-020-2141-2 (PMC7093969; doi:10.1186/s13075-020-2141-2)
Supplement: Supplementary file 5 — Additional file 5: Table S2. Hazard ratios for renal crisis from a multivariable Cox proportional hazard model with covariates measured at baseline based on the medication dataset. [file 13075_2020_2141_MOESM5_ESM.docx]

|  | No. of renal crises/patients | Hazard ratio (95% CI) | P value |
| --- | --- | --- | --- |
| Age (per decade) | 70/4972 | 1.02 (0.83 - 1.25) | 0.84 |
| Sex (male) |  | 1.38 (0.76 - 2.50) | 0.29 |
| Diffuse skin involvement |  | 1.88 (1.07 - 3.32) | 0.028 |
| Time since onset of scleroderma (per decade) |  | 0.88 (0.63 - 1.22) | 0.43 |
| Arterial hypertension |  | 2.31 (1.34 - 3.97) | 0.003 |
| Tendon friction rub |  | 1.74 (0.84 - 3.58) | 0.14 |
| ACE inhibitors |  | 2.02 (1.20 - 3.41) | 0.009 |
| SCL 70 positive |  | 1.28 (0.73 - 2.25) | 0.40 |
| ACA positive |  | 1.11 (0.59 - 2.11) | 0.74 |
| Glucocorticoids > 10mg |  | 1.12 (0.40 - 3.12) | 0.83 |
| PDE5 inhibitors |  | 1.84 (0.73 - 4.63) | 0.19 |
