# Supplementary material for: ACE inhibitors in SSc patients display a risk factor for scleroderma renal crisis—a EUSTAR analysis
Source: Arthritis Res Ther. 2020 Mar 24;22:59. doi: 10.1186/s13075-020-2141-2 (PMC7093969; doi:10.1186/s13075-020-2141-2)
Supplement: Supplementary file 6 — Additional file 6: Table S3. Hazard ratios for renal crisis from a multivariable Cox proportional hazard model with covariates observed at any time before renal crisis based on the medication dataset. [file 13075_2020_2141_MOESM6_ESM.docx]

|  | No. of renal crises/patients | Hazard ratio (95% CI) | P value |
| --- | --- | --- | --- |
| Age at baseline | 78/6083 | 1.08 (0.89 - 1.32) | 0.42 |
| Sex (male) |  | 1.34 (0.77 - 2.34) | 0.31 |
| Diffuse skin involvement |  | 1.41 (0.84 - 2.39) | 0.19 |
| Time since onset of scleroderma (per decade) |  | 0.76 (0.55 - 1.07) | 0.12 |
| Arterial hypertension |  | 1.55 (0.92 - 2.62) | 0.10 |
| Tendon friction rub |  | 0.97 (0.54 - 1.73) | 0.92 |
| ACE inhibitors |  | 2.03 (1.23 - 3.33) | 0.005 |
| SCL 70 positive |  | 0.91 (0.55 - 1.52) | 0.73 |
| ACA positive |  | 0.70 (0.40 - 1.21) | 0.20 |
| Glucocorticoids > 10mg |  | 1.25 (0.59 - 2.64) | 0.55 |
| PDE5 inhibitors |  | 0.98 (0.49 - 1.98) | 0.96 |
