# Supplementary material for: ACE inhibitors in SSc patients display a risk factor for scleroderma renal crisis—a EUSTAR analysis
Source: Arthritis Res Ther. 2020 Mar 24;22:59. doi: 10.1186/s13075-020-2141-2 (PMC7093969; doi:10.1186/s13075-020-2141-2)
Supplement: Supplementary file 8 — Additional file 8: Table S5. Subhazard ratios for renal crisis from a multivariable competing risk model with death (without SRC) as competing event based on the medication dataset. [file 13075_2020_2141_MOESM8_ESM.docx]

|  | No. of renal crises/patients | Hazard ratio (95% CI) | P value |
| --- | --- | --- | --- |
| Age (per decade) | 78/6083 | 1.03 (0.85 - 1.24) | 0.78 |
| Sex (male) |  | 1.25 (0.70 - 2.25) | 0.45 |
| Diffuse skin involvement |  | 1.71 (0.99 - 2.96) | 0.05 |
| Time since onset of scleroderma (per decade) |  | 0.76 (0.51 - 1.14) | 0.19 |
| Arterial hypertension |  | 2.28 (1.36 - 3.81) | 0.002 |
| Tendon friction rub |  | 1.71 (0.85 - 3.43) | 0.13 |
| ACE inhibitors |  | 2.07 (1.27 - 3.38) | 0.004 |
| SCL70 positive |  | 0.98 (0.59 - 1.61) | 0.92 |
| ACA positive |  | 0.84 (0.46 - 1.50) | 0.55 |
| Glucocorticoids > 10mg |  | 1.42 (0.51 - 3.92) | 0.50 |
| PDE5 inhibitors |  | 1.23 (0.56 - 2.74) | 0.61 |
