# Supplementary material for: ACE inhibitors in SSc patients display a risk factor for scleroderma renal crisis—a EUSTAR analysis
Source: Arthritis Res Ther. 2020 Mar 24;22:59. doi: 10.1186/s13075-020-2141-2 (PMC7093969; doi:10.1186/s13075-020-2141-2)
Supplement: Supplementary file 9 — Additional file 9: Table S6. Hazard ratios for the effect of ACEi on SRC from Cox proportional hazard models adjusted for age, sex, disease severity, and time since onset of scleroderma at baseline, and arterial hypertension, tendon friction rub, SCL-70, ACA, glucocorticoids >10mg and PDE5 inhibitors measured at baseline or at any time before renal crisis using different propensity score methods, i.e. one-to-one matching, k-nearest neighbors matching and inverse probability weighting. [file 13075_2020_2141_MOESM9_ESM.docx]

|  | No. of renal crises/patients | Hazard ratio (95% CI) | P value |
| --- | --- | --- | --- |
| **Covariates measured at baseline** |  |  |  |
| One-to-one matching | 39/1780 | 2.01 (1.03 - 3.93) | 0.041 |
| 3-nearest neighbors matching | 50/2483 | 2.10 (1.15 - 3.84) | 0.016 |
| 5-nearest neighbors matching | 53/3014 | 1.75 (0.95 - 3.22) | 0.07 |
| 10-nearest neighbors matching | 61/3801 | 1.83 (1.04 - 3.25) | 0.037 |
| Inverse probability weighting | 68/4927 | 2.39 (1.37 - 4.18) | 0.002 |
| **Covariates at any time before SRC** |  |  |  |
| One-to-one matching | 56/3164 | 1.82 (1.05 - 3.16) | 0.032 |
| 3-nearest neighbors matching | 64/3907 | 1.68 (0.94 - 3.01) | 0.08 |
| 5-nearest neighbors matching | 73/4448 | 1.74 (1.04 - 2.91) | 0.036 |
| 10-nearest neighbors matching | 75/5175 | 1.70 (1.01 - 2.84) | 0.045 |
| Inverse probability weighting | 74/5997 | 2.20 (1.33 - 3.64) | 0.002 |
